# A Policy Analysis of Biodiversity Offsetting: Benchmarking Against International Best Practice Principles

**DOI:** 10.1007/s00267-025-02350-8

**Published:** 2025-12-27

**Authors:** Rocío A. Cares, Alan Bond, Aldina M. A. Franco

**Affiliations:** 1 School of Environmental Sciences, University of East Anglia (UEA), Norwich, England UK; 2 Research Unit for Environmental Sciences and Management, North-West University, Potchefstroom, South Africa

**Keywords:** Biodiversity offset, Compensation of biodiversity, Chile, Best practice principles, Benchmark

## Abstract

Given global threats to biodiversity, implementing effective biodiversity offset policies is increasingly recognised as being essential for delivering sustainable development. As research and practice on offsets has developed, so have international expectations of best practice principles, which set the benchmark for national systems in their efforts to protect biodiversity. This research aims to synthesise best practice principles for biodiversity offsets from the international literature, developing a benchmark to assess the extent to which national policies align with international standards. Chile is selected as a suitable case study due to its biodiversity richness and emerging biodiversity offsets policy, to test this analytical framework. The analysis indicates that the benchmark provides a useful basis for assessing national biodiversity offset policies and shows that Chilean policy demonstrates an initial alignment with international best practices, though several areas for improvement remain.

## Introduction

Biodiversity refers to the variety of living organisms across all sources, encompassing genetic diversity within species, differences between species, and the range of ecosystems (CBD 1992). Biodiversity offsetting involves compensating for ecological losses by creating ecological gains through measures such as ecological restoration, the creation of new protected areas, or various forms of habitat management (zu Ermgassen et al. 2019). The overall concept is that development projects should lead to ‘no net loss’ and even achieve a ‘net gain’ or ‘net positive impact’ on biodiversity (Moilanen and Kotiaho 2021).

Biodiversity offsetting has been a common practice since at least the 1970s in Europe (Damiens et al. 2021a), with the practice spreading globally through the adoption of a diverse range of governance approaches to biodiversity offsetting (GIBOP 2019). This evolution has been driven by the collaborative efforts of various stakeholders, including governments, NGOs, businesses, and academia (Souza et al. 2023). Policy advocates such as the Business and Biodiversity Offsets Programme (BBOP) have established standards and biodiversity offset mechanisms for projects that draw on the terminology and experiences of members (Damiens et al. 2021a).

In recent decades, biodiversity considerations have become integral to Environmental Impact Assessment (EIA) processes worldwide. For instance, the EU’s EIA Directive (2014/52/EU) explicitly requires the assessment of biodiversity impacts and the identification of compensation measures. Similarly, Australia and Canada have incorporated biodiversity within their EIA frameworks to support global biodiversity targets (Wegner et al. 2005; Gannon 2021). In developing countries, Brazil and South Africa have embedded biodiversity considerations in EIA and spatial planning (Ritter et al. 2017; Swanepoel et al. 2019). Recognising the critical importance of ecosystems services and biodiversity for sustaining life and mitigating climate change, many countries have adapted their EIA frameworks to include mechanisms for biodiversity offsetting and goals for no net loss (Condon et al. 2020; Damiens et al. 2021a; Cares et al. 2023; Souza et al. 2023; Morrison-Saunders and Sánchez 2024; Ghijselinck et al. 2026). This growing emphasis reflects a collective response to unprecedented rates of biodiversity loss due to human activities, as highlighted by various international frameworks and standards, including the World Bank (2016), the International Finance Corporation Performance Standard 6 (IFC 2019), the Kunming-Montreal Global Biodiversity Framework (GBF) adopted in 2022 by the Parties to the UN Convention on Biological Diversity (CBD 1992), the EU’s biodiversity strategy 2030, and the UK Biodiversity Net Gain (BNG). These policies reflect an increasing trend to incorporate biodiversity offsets into regulatory frameworks, particularly for projects located in ecologically sensitive areas.

Biodiversity offsets have increasingly been framed within the broader context of sustainable development, as they seek to reconcile economic growth with the conservation of natural capital (Abdo et al. 2019). However, the use of biodiversity offsets remains controversial. Critics argue that offsets may legitimize environmental degradation or fail to deliver equivalent ecological outcomes, especially where ecological equivalence is difficult to achieve or monitor (Lindenmayer et al. 2017; Apostolopoulou and Adams 2017; Vardon et al. 2025; Maron et al. 2025). Recognizing these challenges is essential to ensure that biodiversity offsets function as a complementary tool within a wider sustainability framework, rather than as a substitute for avoidance and mitigation measures (Cares et al. 2023; Ghijselinck et al. 2026).

This paper aims to synthesise best practice principles for biodiversity offsets from the international literature, developing a benchmark to assess the extent to which national policies align with international standards. To achieve this aim, two objectives are established:

1. to undertake a comprehensive synthesis of international best practice principles on biodiversity offsetting for development projects, in order to establish a benchmark for policy assessment;
2. to apply this benchmark to evaluate the extent to which a selected national policy aligns with these international standards.

By outlining current international best practice expectations for biodiversity offsets, this paper offers a clear benchmark to assess and guide the development or evaluation of national biodiversity offset systems. Chile is selected as an appropriate national policy for managing the biodiversity implications of projects against which to test this benchmark, with the next section justifying this selection. Section “Methods” sets out the methods for developing and operationalise the benchmark for policy comparison. Section “Results” tests Chilean biodiversity offsetting policy against the benchmark before Section “Discussion” discusses the results and concludes.

## Biodiversity Offset Policy in Chile

Chile is renowned for its high biodiversity and endemism, shaped by unique biogeographic conditions (MMA 2019). It hosts diverse ecosystems, terrestrial, marine, coastal, and oceanic islands, that are vital for economic development, social well-being, and ecosystem services (Lara et al. 2009). Chile is home to one of five global Mediterranean-climate regions (McNally 1990), the Chilean Winter Rainfall-Valdivian Forest, a global biodiversity hotspot (Mittermeier et al. 2011), and 88 of the planet’s 110 ecosystems (Keith et al. 2022). These ecosystems are under significant threat from activities such as mining, agriculture, and urban expansion (Padhiary and Kumar 2024). Consequently, efforts to integrate biodiversity considerations into EIA processes have continued to advance, strengthening their role in supporting project decision-making (Mandai and de Souza 2021).

In Chile, a regulatory framework for biodiversity offsets (known as ‘appropriate compensation of biodiversity’ in Chile (Cares et al. 2023)) is embedded within the Environmental Impact Assessment System (EIAS) and is continually evolving. In 2014 the first *Guía para la compensación de biodiversidad en el SEIA* [Guide for Biodiversity Compensation in the EIAS] was published by the *Servicio de Evaluación Ambiental* [Environmental Assessment Service] (SEA 2014), which detailed the minimum essential elements required for appropriate compensation for biodiversity loss. The Guide specified that the negative impacts on biodiversity identified by the developer must be balanced by a positive effect, hence development projects or activities indicated in the Law N°19,300 on *Bases Generales del Medio Ambiente* [General Environmental Bases], must promote at least a zero net loss of biodiversity, or even a net gain (SEA 2014). This national guide, which standardises criteria, requirements, conditions, and technical specifications for implementing appropriate biodiversity compensation, as well as ensuring adherence to the regulatory framework in Chile, was updated in 2022 to reduce the scope for discretionary decision-making (SEA 2022). This update is consistent with the guidelines set out in the National Biodiversity Strategy (NBS) 2017–2030, which is the public policy instrument that establishes the main strategic guidelines and national targets for the conservation and sustainable use of biodiversity up to 2030 (MMA 2018). The NBS implements the commitments to the Convention on Biological Diversity (CBD) based on Chile being a signatory country since 1994. Additionally, responding to the need to establish a single methodology for the design and implementation of biodiversity offsetting measures, the first edition of the *Guía metodológica para la compensación de la biodiversidad en ecosistemas terrestres y acuáticos continentales* [Methodological guide for the compensation of biodiversity in terrestrial and inland aquatic ecosystems] was published in 2022, establishing a specific and comprehensive methodology for the design and implementation of biodiversity compensation measures in terrestrial and inland aquatic ecosystems. A second edition of this Guide was published in 2023 to provide developers with new technical specifications that facilitate the practical application of the methodology (SEA 2023a). These Guides are binding on the EIA process, and demand that the design and methodology of compensation measured should be in line with the requirements set out in these guidelines.

## Methods

A benchmark was developed based on internationally recognised best practice principles for biodiversity offsetting. The process involved the following steps:

### Literature Selection

A literature review was conducted to identify and synthesise international best practice principles for biodiversity offsets, since a thorough review consolidates prior research and supports theory development (Snyder 2019). The review focused on international guidance documents and published literature on best practice principles for biodiversity offsetting. While integrating biodiversity offset strategies into the EIA process is increasingly common (de Witt et al. 2019; Pope et al. 2021), the review aimed to identify key principles for effective biodiversity offset planning, applicable to both conservation outcomes and impact assessments.

While some researchers conceptualise practice elements as goals, emphasizing their aspirational nature in guiding conservation outcomes (Maron et al. 2016; Souza et al. 2023), others argue that these elements function as principles, providing structured and normative guidance that informs the implementation of offsetting in a consistent and transparent manner (Bull and Brownlie, McKenney and Kiesecker 2010; Chee 2015; 2017; Brownlie and Treweek 2018; Maron et al. 2018). This paper adopts the term ‘principles’ to describe best practice elements, aligning with the approach taken by the Business and Biodiversity Offsets Programme (BBOP 2012b; 2018), which defines principles as fundamental rules that underpin effective biodiversity offsetting and which build on their original ten principles published in 2009 (BBOP 2009).

The research relied on Scopus and Google Scholar, two of the largest literature databases. Scopus, known for its broad coverage (Burnham 2006; Waltman 2016), was complemented by Google Scholar, which provides diverse results compared to Scopus (Bar-Ilan 2008), ensuring a more comprehensive and varied literature review.

Following the approach outlined by Aromataris and Riitano (2014), a search string was developed through an iterative process of refinement, focusing on the research question and its key terms, resulting in a logical concept map. To identify best practice principles for biodiversity offsets, the following search strings were applied to both databases:

- (“Biodiversity Offset” OR “Environmental Offsets” OR “offsets”) AND (“best practice” OR principles OR policy)
- (“No net loss” OR “NNL” OR “zero net loss”) AND (“best practice” OR principles OR policy)
- (“Biodiversity net gain” OR “BNG”) AND (“best practice” OR principles OR policy)

To ensure comprehensive coverage of relevant literature, the search strategy included variations of key terms related to biodiversity offsets. In addition to the term “biodiversity offsets”, we also considered “environmental offsets” and “offsets” as search terms, given that terminology varies across jurisdictions and policy contexts. Best practices and principles were included to encompass all the values and fundamental guidelines discussed in the literature that influence how biodiversity offsetting should ideally be carried out. This broad inclusion ensures that all types of guiding concepts, whether theoretical principles or practical best practices, are captured. Additionally, policy was also included in the search to make sure that any literature discussing formal rules, procedures, and legal frameworks for biodiversity offsetting was also reviewed. This approach ensures that sources related to regulatory requirements and structured approaches to offset implementation are included, distinguishing between high-level guiding principles and actionable policy measures that enforce biodiversity offsets in practice.

After conducting database searches, the results were examined to determine inclusion or exclusion based on their relevance. The rationale for selecting papers was driven by the need to evaluate the alignment of national policies with international principles and practices. Therefore, the review included papers that:

- Address multiple offsetting principles, even if they concentrated on specific aspects, to guide policies or practice.
- Discuss practical or theoretical aspects of implementation (and policy-making) of the biodiversity offset.
- Contribute to understanding the broader landscape of offset policy and practice.

The inclusion criteria were as follows: (i) materials published in English, (ii) peer-reviewed publications (such as articles and book chapters) as well as grey literature (such as studies and guidance documents from federal agencies or non-government entities such as BBOP), (iii) works published between 2012 and 2024 to capture recent developments in the field, starting with the release of the first Standard on Biodiversity Offsets by BBOP in 2012 (including their 2009 Principles, BBOP (2009)), and (iv) literature that emphasised principles, policy, or practice in biodiversity offsetting. Literature analysing biodiversity offsetting from a purely methodological or scientific perspective, without reference to principles, policy, or practice, was excluded to maintain relevance to the research scope. Additionally, works that referenced the BBOP principles (including those published in 2009) or similar sources without further development of their principles were also excluded to avoid duplication of information.

The search process was initially filtered by titles and keywords, followed by a second filter based on reading abstracts, excluding articles that did not meet the inclusion criteria. Full texts of the selected articles were then reviewed, with a snowballing approach (Wohlin 2014) used to identify additional relevant references. This involved examining references within key articles and tracking citing articles through citation indices, ensuring comprehensive coverage of relevant literature.

**Fig. 1:**
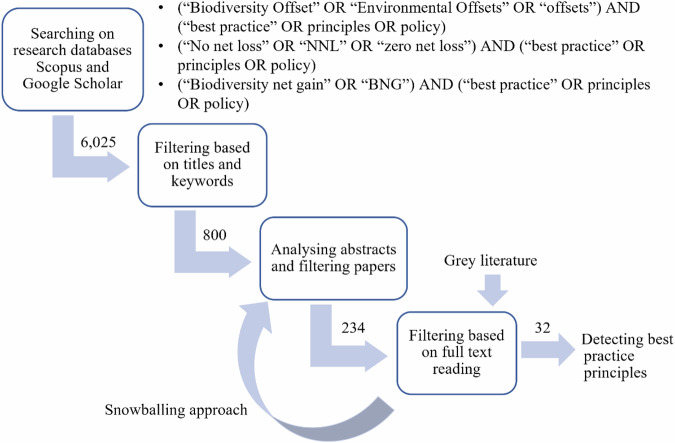
Flowchart of the methodology for detecting the international best practice guidance from the literature review

Grey literature was identified through targeted online searches and manual screening of publications from organisations actively involved in biodiversity offsetting policy and practice. Organisations were considered relevant based on their recognised role in developing, implementing, or advising on biodiversity offset frameworks. Search strategies involved using combinations of key terms (“biodiversity offset,” “no net loss,” “mitigation hierarchy”, “net gain”) with organisation names to identify relevant documents such as policy reports, guidelines, and technical papers. Relevance was assessed based on whether the publication explicitly addressed biodiversity offsetting principles, provided practical guidance on implementation, or informed policy and regulatory frameworks related to no net loss or mitigation measures. Six guidelines were included, selected for their direct relevance to biodiversity offsetting and their contribution to understanding practical applications and implementation challenges. The process resulted in a final selection of 32 articles. Figure 1 illustrates the methodology used to identify best practice principles for biodiversity offsetting, and report the number of records retrieved, screened and retained at each stage of the process.

### Extraction of the Principles

Following the literature selection, a systematic process was undertaken to extract relevant biodiversity offsetting principles from each source. This involved a detailed reading of each document to identify passages where best practice standards or guiding principles were explicitly stated or implicitly discussed. The extraction process included both deductive and inductive approaches: (i) Deductive extraction focused on identifying principles that were explicitly labelled in the literature. These were often found in policy guidance documents or academic reviews that listed and defined best practice principles. (ii) Inductive extraction involved identifying principles that were not explicitly labelled but were clearly discussed in the context of good practice. For example, some sources elaborated on the importance of early offset planning, or on addressing cumulative impacts, without labelling these as formal “principles”. These discussions were thematically coded and included in the list of extracted principles where they reflected consistent and significant themes across sources.

During this process, a matrix was constructed in Excel™ in which each row represented a source (academic or grey literature), and each column corresponded to an identified principle. For each cell, the associated text or definition of the principle was entered, along with a citation to its source. This enabled cross-comparison across literature and supported the identification of overlapping or closely related principles. Through thematic analysis (Ward et al. 2009) principles with equivalent or closely aligned definitions were consolidated into 16 overarching principles, allowing conceptual clarity, avoiding redundancy, and maintaining consistency in terminology based on existing usage in the literature. These principles collectively formed the benchmark for what constitutes international best practice in biodiversity offsetting.

### Translation into Guiding Questions

To operationalise the benchmark for policy comparison, each of the 16 principles was translated into one or two guiding questions. These questions were designed to support systematic document analysis, ensuring that each principle could be consistently and transparently assessed against biodiversity offsetting national policies.

The development of guiding questions followed a deductive logic, drawing from thematic coding techniques common in qualitative policy analysis (Braun and Clarke 2006; Bowen 2009). For each principle, its core conceptual components were identified and translated into specific, answerable questions (Babbie 2020). These questions were phrased to reflect both the intent and operational conditions of each principle, allowing them to function as analytical criteria during document analysis (Ritchie and Spencer 2002; Bowen 2009).

### Application to Policy Review

This benchmark served as the analytical foundation for evaluating the Chilean policy framework. The use of clearly defined guiding questions enabled a systematic and repeatable approach to identifying the extent to which each principle was reflected or absent in national policy documents. The current Chilean policy for biodiversity offsets comprises the following guide:

*Guía para la compensación de biodiversidad en el SEIA* [Guide for Biodiversity Compensation in the EIAS] (SEA 2022). Additionally, this national guide was complemented with the *Guía metodológica para la compensación de la biodiversidad en ecosistemas terrestres y acuáticos continentales* [Methodological guide for the compensation of biodiversity in terrestrial and inland aquatic ecosystems] (SEA 2023a) and the *Guía para la Participación Ciudadana Temprana en proyectos que se presentan al Sistema de Evaluación de Impacto Ambiental* [Guide for Early Citizen Participation in projects submitted to the Environmental Impact Assessment System] (SEA 2023b). Reference to the National Guides in the following text includes the content of the two complementary guides on compensation methodology and citizen participation.

The National Guides were developed based on the principles established by the Business and Biodiversity Offsets Programme established in 2009 (BBOP 2009), as outlined in those guides (SEA 2022), but adapted to the Chilean context.

## Results

### Overview of Benchmark Principles and Guiding Questions

To develop the benchmark used for policy comparison, best practice principles were identified through a systematic review of international literature. These sources include standards, policy guidelines, and peer-reviewed studies that have been influential in shaping global approaches to biodiversity offsetting. The studies were summarised by geographical distribution (whether biodiversity offsets in each jurisdiction operate under mandatory, voluntary, or mixed regulatory frameworks), type of documents included (peer-reviewed, book chapter and grey literature) to illustrate the diversity of evidence supporting the analysis, and whether the publications addressed interactions between biodiversity offset principles and other conservation or market-based mechanisms (such as biodiversity markets, EIA, land-use planning, social offsets, among others), to contextualise how offset principles function within broader environmental governance arrangements. These details are synthesised in Table 1, whilst Table 2 summarises the documents included in the review and the specific principles derived from each. This process ensured that the benchmark was grounded in authoritative and diverse sources, providing a robust reference for evaluating Chilean policy.

**Table 1:** Overview of the literature included in the review

Author	Country/region	Mandatory offsets?	Type of document	Interaction with other conservation mechanisms?
Abdo et al. (2019)	Global	Not applicable	Peer-reviewed	Linked to sustainable development
Benabou (2014)	Global	Not applicable	Peer-reviewed	Discuss market-based instruments
Bergès et al. (2020)	France	Yes	Peer-reviewed	No
Bidaud et al. (2018)	Madagascar	Voluntary	Peer-reviewed	Linked to social offsets
Brown and Penelope (2016)	New Zealand	Yes	Peer-reviewed	No
Brownlie and Treweek (2018)	Global	Not applicable	Grey literature	Applicable to EIA
Brunetti et al. (2023)	France	Yes	Peer-reviewed	No
Bull et al. (2017)	Global	Not applicable	Peer-reviewed	No
Bull and Brownlie (2017)	Global	Not applicable	Peer-reviewed	No
Bull and Strange (2018)	Global	Not applicable	Peer-reviewed	No
BBOP (2018)^a^	Global	Voluntary	Grey literature	No
Chee (2015)	Global	Not applicable	Book chapter	No
CIEEM (2016)	UK	Yes	Grey literature	No
Damiens et al. (2021b)	Global	Not applicable	Peer-reviewed	Discuss polluter-pays principle
de Witt et al. (2019)	South Africa	Yes	Peer-reviewed	No
Evans (2023)	Australia	Yes	Peer-reviewed	No
Fallding (2014)	Australia	Yes	Peer-reviewed	Linked to land-use planning processes
Fitzsimons et al. (2014)	Australia	Yes	Peer-reviewed	No
Grimm and Köppel (2019)	Global	Not applicable	Peer-reviewed	No
IFC (2019)	Global	Mandatory for IFC-financed projects	Grey literature	Linked to sustainable development
IUCN (2016)	Global	Voluntary	Grey literature	Linked to broader conservation planning tools
Jacob et al. (2020)	Global	Not applicable	Peer-reviewed	No
Maron et al. (2021)	Global	Not applicable	Peer-reviewed	Linked to the GBF
Middle and Middle (2010)	Australia	Yes	Peer-reviewed	Applicable to EIA
Niner et al. (2017)	Global	Not applicable	Peer-reviewed	Linked to biodiversity markets
Quétier et al. (2014)	France	Yes	Peer-reviewed	No
Salès et al. (2023a)	Peru and France	Yes	Peer-reviewed	No
Salès et al. (2023b)	Colombia and France	Partial/Yes	Peer-reviewed	Linked to land-use planning instruments
Simmonds et al. (2022)	Global	Not applicable	Peer-reviewed	Linked to the GBF
Souza et al. (2023)	Brazil	Yes	Peer-reviewed	No
Vardon et al. (2025)	Australia	Yes	Peer-reviewed	Linked to natural capital
WBG (2016)	Global	Voluntary	Grey literature	Discuss conservation finance

^a^Given BBOP (2018) postdates the BBOP 2009 principles, the latter were not included.

**Table 2:** Sources informing the benchmark of international best practice principles for biodiversity offsetting

Authors/principles	Adherence to the mitigation hierarchy	Biodiversity net gain (BNG)	Limits to what can be offset	Additionality	Equivalence/like-for-like	Proportionate in size and scale	Offsets from earliest stages	Long-term outcomes	Precautionary approach	Ecosystem approach	Adaptive management and monitoring	Cumulative, direct and indirect impacts	Compliance with monitoring and enforcement	Participatory and transparent approach	Support evidence-based approaches	Equity and rights-based approach
Abdo et al. (2019)		x		x	x	x	x	x		x	x		x			
Benabou (2014)	x			x	x											
Bergès et al. (2020)	x															
Bidaud et al. (2018)																x
Brown and Penelope (2016)	x		x		x								x			
Brownlie and Treweek (2018)	x						x		x	x	x			x	x	
Brunetti et al. (2023)	x				x											
Bull et al. (2017)				x												
Bull and Brownlie (2017)		x														
Bull and Strange (2018)	x															
BBOP (2018)^a^	x		x	x				x		x				x	x	x
Chee (2015)	x		x						x				x			
CIEEM (2016)	x	x	x	x				x	x					x		x
Damiens et al. (2021b)				x	x			x			x		x	x		
de Witt et al. (2019)	x	x	x	x	x		x	x	x	x		x	x	x		
Evans (2023)				x		x			x				x	x		
Fallding (2014)	x				x		x	x	x				x	x	x	x
Fitzsimons et al. (2014)	x			x		x		x	x			x		x	x	
Grimm and Köppel (2019)					x			x	x	x	x		x	x		
IFC (2019)				x	x					x						
IUCN (2016)			x	x			x		x	x		x	x		x	x
Jacob et al. (2020)			x	x	x			x						x		
Maron et al. (2021)			x		x			x		x						
Middle and Middle (2010)	x				x	x		x					x	x		
Niner et al. (2017)				x	x			x					x			
Quétier et al. (2014)				x			x	x			x					
Salès et al. (2023a)	x			x	x											
Salès et al. (2023b)	x			x	x											x
Simmonds et al. (2022)		x							x							
Souza et al. (2023)			x	x	x		x	x	x	x	x			x		
Vardon et al. (2025)	x			x	x											
WBG (2016)				x	x			x								

^a^Given BBOP (2018) postdates the BBOP (2009) principles, the latter were not included

The following section presents the benchmark rationale for each principle alongside the guiding question it informed. This format provides transparency regarding how international standards were interpreted and applied for evaluating the Chilean policy framework.

#### Principle 1. Adherence to the mitigation hierarchy

##### Benchmark rationale

The review of literature identified that adherence to the mitigation hierarchy is a fundamental principle in biodiversity offsetting aimed at minimising the negative effects of development on biodiversity (BBOP 2018). This sequential approach mandates that attempts should be made to first avoid impacts through preventive measures and alternative project designs, applied broadly in environmental impact assessment (Brownlie and Treweek 2018; de Witt et al. 2019; Bergès et al. 2020). When complete avoidance is not feasible, steps must be taken to minimise and reduce impacts as much as possible, followed by on-site rehabilitation or restoration efforts (Fitzsimons et al. 2014; Brunetti et al. 2023). Only after these measures have been thoroughly pursued should biodiversity offsets be considered, as a last resort, to compensate for significant residual impacts (Niner et al. 2017). Residual impacts are the adverse effects on biodiversity that remain after all reasonable measures to avoid, minimise, and rehabilitate impacts have been fully applied in accordance with the mitigation hierarchy (Benabou 2014).

##### Guiding questions

1.a) Does the policy explicitly reference the full mitigation hierarchy (avoid, minimise, restore, offset)?

1.b) Does the policy require demonstrating that avoidance and minimization have been fully considered before offsetting?

#### Principle 2. Biodiversity Net Gain (BNG)

##### Benchmark rationale

BNG refers to an approach to biodiversity conservation where developments leave biodiversity in a measurably better state than before the project began (CIEEM 2016). It goes beyond the principle of no net loss (NNL), which aims to quantify and balance biodiversity losses from development with equivalent gains elsewhere (BBOP 2018; IFC 2019). While BNG is often promoted as a positive objective, its value depends on how it is defined and measured, ensuring that the total biodiversity is enhanced as a result of human activities (Moilanen and Kotiaho 2021). Biodiversity offsets only deliver positive outcomes when they incorporate ecological effectiveness, landscape-level considerations, social aspects, and clear requirements for transparency, measurability and enforcement (Abdo et al. 2019). NNL has long been recognised as a core principle for biodiversity offsetting and was the starting point for this research, with many authors promoting NNL as the minimum standard and BNG as the preferred goal (Fallding 2014; Quétier et al. 2014; IUCN 2016; Brownlie and Treweek 2018; BBOP 2018; Salès et al. 2023b). However, growing evidence shows that NNL alone is not enough to address the scale of global biodiversity decline, indicating that more ambitious and ecologically robust approaches are needed (Gibbons and Lindenmayer 2007; Bull and Brownlie 2017; Maron et al. 2018; Moilanen and Kotiaho 2018; Maron et al. 2020). Therefore, a shift toward BNG is not only desirable but necessary.

##### Guiding questions

2.a) Is BNG defined in the policy, and if so, how is it defined?

2.b) Does it specify metrics or methods to demonstrate whether net gain is achieved?

#### Principle 3. Limits to what can be offset

##### Benchmark rationale

Best practice biodiversity offsets should incorporate the principle of limits to what can be offset (Chee 2015; BBOP 2018; de Witt et al. 2019). Biodiversity offsets should not be allowed in situations involving rare, unique, or threatened species or ecosystems, or when the species or communities have special cultural or economic importance (Abdo et al. 2019). For irreplaceable or vulnerable values of biodiversity, no loss instead of no net loss should be the requirement (Maron et al. 2021). Limits also apply in situations where implementing offsets may not be feasible due to legal, financial, institutional, or sociocultural constraints (BBOP 2012a).

##### Guiding questions

3.a) Are there criteria or thresholds defined to determine when biodiversity loss is unacceptable and cannot be offset?

3.b) Does the policy provide criteria or guidance to assess when offsets are not feasible or appropriate, including in cases of major uncertainty or high risk?

#### Principle 4. Additionality

##### Benchmark rationale

Biodiversity offsets must deliver conservation outcomes that are above and beyond what would have occurred without the offset (Souza et al. 2023). This requires delivering measurable net gains for biodiversity that exceed existing obligations, legal requirements, or ongoing conservation activities (Fitzsimons et al. 2014; Quétier et al. 2014). Offsets must provide additional benefits, which means that the gains from the offsets should exceed the losses, and biodiversity offsets must generate conservation outcomes that go beyond the results expected without their implementation (Niner et al. 2017; de Witt et al. 2019; Jacob et al. 2020; Evans 2023).

##### Guiding question

4.a) Does the policy require biodiversity offsets to deliver conservation outcomes beyond those already mandated?

#### Principle 5. Equivalence/like-for-like

##### Benchmark rationale

The review identified that one of the most important principles of biodiversity best practice is equivalence (Benabou 2014). Offsets should ensure ecological equivalence and to generate gains that are equivalent to, and thus compensatory for, the ecological losses incurred by development projects (Abdo et al. 2019; Fitzsimons et al. 2014; Maron et al. 2021). Offsets must adhere to the like-for-like or better standard (Fallding 2014; de Witt et al. 2019; IFC 2019), and aim to conserve the same environmental values that are being affected (i.e., “in-kind” offsets) (Salès et al. 2023b). However, in instances where the impacted areas are deemed to hold little conservation value, “out-of-kind” offsets may be considered (Benabou 2014).

##### Guiding question

5.a) Does the policy require offsets to compensate for the full suite of natural and environmental values affected by the project?

#### Principle 6. Proportionate in size and scale

##### Benchmark rationale

Biodiversity offsets should be proportionate in size and scale to the residual impacts on the affected environmental values (Evans 2023). This proportionality ensures that the offset effectively addresses the extent and severity of ecological damage caused by development projects (Fitzsimons et al. 2014).

##### Guiding questions

6.a) Does the policy require that the size and scale of biodiversity offsets be proportionate to the residual impacts?

6.b) Does the policy provide guidance or criteria for determining that proportionality?

#### Principle 7. Offsets from earliest stages

##### Benchmark rationale

Integrating biodiversity and ecosystem services into development planning and EIA should commence at the earliest stages of project development to guide sustainable decision-making (Brownlie and Treweek 2018). Offsets must be established before any activities that could cause biodiversity loss begin (Fallding 2014; de Witt et al. 2019), ensuring that suitable, direct offsets, and potentially other compensatory measures, are in place (Fallding 2014; Evans 2023). Direct offsets refer to conservation actions that generate measurable biodiversity gains of the same type, at the same scale, and for the same ecological features that are adversely affected by a project, and in contrast, indirect offsets (or compensatory measures) involve actions that support biodiversity more broadly, such as knowledge acquisition and research funding, but do not directly replace the specific biodiversity values lost (Abdo et al. 2019). Offset measures should be timely and structured to achieve biodiversity gains as promptly as possible, ideally before the associated losses occur (Maron et al. 2021; Souza et al. 2023). This approach helps prevent irreversible damage and mitigates the potential time lag between the occurrence of impacts and the realisation of offset benefits (Quétier et al. 2014).

##### Guiding question

7.a) Does the policy require that biodiversity offsets will be considered and planned from the earliest stages of project design and decision-making?

#### Principle 8. Long-term outcomes

##### Benchmark rationale

Best practice principles in biodiversity offsets should incorporate the principle of long-term outcomes (BBOP 2018; de Witt et al. 2019; Souza et al. 2023). Biodiversity offsets must be designed to endure for as long as the residual impacts of development occur (Abdo et al. 2019; Fallding 2014), and the benefits of offsets must be delivered for the duration of these impacts, ideally in perpetuity (WBG 2016; Grimm and Köppel 2019), focusing on achieving long-term strategic outcomes (Fitzsimons et al. 2014).

##### Guiding questions

8.a) Does the policy require monitoring of offset outcomes over the long term?

8.b) Does the policy ensure biodiversity gains are maintained beyond the project duration?

#### Principle 9. Precautionary approach

##### Benchmark rationale

A decision-making principle that requires taking preventive action to avoid or minimise harm when there is scientific uncertainty about potential environmental impacts (Brownlie and Treweek 2018; de Witt et al. 2019). Under this approach, lack of full knowledge or incomplete data is not a justification for delaying measures to protect biodiversity; instead, decisions should err on the side of caution to safeguard ecosystems, species, and ecological processes (Chee 2015; CIEEM 2016; Evans 2023). The principle ensures that lack of full scientific certainty does not lead to decisions that could cause irreversible biodiversity loss (Fitzsimons et al. 2014; Simmonds et al. 2022).

##### Guiding question

9.a) Does the policy require precautionary measures when there is uncertainty about potential biodiversity impacts or offset effectiveness?

#### Principle 10. Ecosystem approach

##### Benchmark rationale

In the specific context of biodiversity offsetting, the literature suggests that best practice should incorporate an ecosystem approach (BBOP 2018). This approach emphasises that biodiversity offsets should align with landscape and ecosystem strategies, integrating the ecosystem perspective throughout all stages of the mitigation hierarchy (IUCN 2016; de Witt et al. 2019; IFC 2019), allowing ecological changes to be assessed at spatial and temporal scales (Brownlie and Treweek 2018). Establishing explicit net outcome goals at multiple levels of biodiversity (ecosystem, species, and genetic diversity) will ensure that all critical aspects of biodiversity are adequately addressed (Maron et al. 2021).

##### Guiding question

10.a) Does the policy promote an ecosystem approach by considering ecological processes, functions, and interconnections beyond individual species or habitats?

#### Principle 11. Adaptive management and monitoring

##### Benchmark rationale

A comprehensive monitoring and evaluation system should be developed, based on clear indicators to track progress and enable corrective actions as needed for achieving NNL (Chee 2015; Souza et al. 2023). Offset measures must have performance-based ecological goals, accompanied by defined protocols to assess both their effectiveness (i.e., whether actions were taken) and efficacy (i.e., whether those actions achieved the desired results) (Quétier et al. 2014). Clearly defining responsibilities and establishing mechanisms for monitoring implementation is essential (Brownlie and Treweek 2018).

##### Guiding question

11.a) Does the policy require the use of adaptive management strategies based on monitoring results?

#### Principle 12. Address cumulative, direct and indirect impacts

##### Benchmark rationale

The literature indicates that cumulative, direct, and indirect impacts should be addressed to effectively manage environmental impacts (de Witt et al. 2019). Comprehensive impact assessments should be conducted that evaluate not only the direct impacts of a project (primary effects of a project that occur at the same time and place as the activity causing them) but also its indirect (occur later in time or farther away in space from the project activity) and cumulative effects (combined effects of a project when added to other past, present, or reasonably foreseeable future actions) (Feldman 2011). This involves analysing how a project may influence surrounding ecosystems, communities, and resources over time (IUCN 2016; de Witt et al. 2019).

##### Guiding question

12.a) Does the policy require consideration of direct, indirect, and cumulative biodiversity impacts in the design and implementation of offsets?

#### Principle 13. Compliance with monitoring and enforcement

##### Benchmark rationale

Oversight and compliance are essential (de Witt et al. 2019). According to Niner et al. (2017), a third party or regulatory body should maintain oversight to ensure adherence to biodiversity offset requirements. Additionally, it is important to identify and implement the necessary legal, institutional, and financial frameworks to ensure the long-term governance of all mitigation actions and offsets (IUCN 2016). This includes ensuring that offsets are enforceable and auditable, documented in sufficient detail, and governed by transparent arrangements that allow for effective measurement, monitoring, and enforcement (Fallding 2014; de Witt et al. 2019). Finally, effective management and governance are imperative to achieve successful biodiversity outcomes (Evans 2023).

##### Guiding questions

13.a) Does the policy include mechanisms that ensure compliance with offset requirements through monitoring, reporting, and enforcement actions?

13.b) Does the policy include mechanisms to ensure compliance with offset requirements through monitoring, reporting, and enforcement actions?

#### Principle 14. Participatory and transparent approach

##### Benchmark rationale

Best practice biodiversity offsetting should incorporate stakeholder rights, values, and dependencies on biodiversity and environmental values for meaningful and fair decision-making, including throughout the EIA process, ensuring that all relevant voices are heard and considered (Brownlie and Treweek 2018). Projects having negative impacts on biodiversity should facilitate effective participation of stakeholders in evaluating, selecting, designing, implementing, and monitoring biodiversity offsets (BBOP 2018). Early engagement is crucial to foster collaboration, build trust, and integrate diverse perspectives into offset strategies (CIEEM 2016; Souza et al. 2023). By involving stakeholders in these processes, benefits can be fairly shared, and project outcomes can align better with community values and needs (Fallding 2014). The literature also reveals that transparency in planning, implementing, and reporting on biodiversity offsets is key (de Witt et al. 2019; Evans 2023). Clear communication regarding the design, implementation, and outcomes of the offset fosters trust among stakeholders and helps to ensure that everyone understands their roles and contributions (Fallding 2014).

##### Guiding questions

14.a) Does the policy promote a participatory and transparent approach by ensuring public access to information?

14.b) Does the policy promote a meaningful stakeholder engagement throughout the offset process?

#### Principle 15. Support evidence-based approaches

##### Benchmark rationale

Offsets should rely on robust environmental information and knowledge to deliver conservation outcomes that are measurable and sustainable (Brownlie and Treweek 2018; BBOP 2018). Science-based approaches that consider both environmental and social impacts—including the effects of mitigation measures on local livelihoods—are essential to developing responsible and effective offset strategies (IUCN 2016). The process of designing and implementing biodiversity offsets should be well-documented, drawing from established ecological principles and scientific rigor (Fallding 2014). Integrating sound science with traditional knowledge, including Indigenous peoples and local communities who have developed collective practices, understandings, and beliefs through generations of interaction with their environment (IFC 2019), ensures that offsets are contextually appropriate and ecologically effective (BBOP 2018).

##### Guiding question

15.a) Does the policy require the use of scientific evidence, data, and best available knowledge to inform biodiversity offset design, implementation, and evaluation?

#### Principle 16. Equity and rights-based approach

##### Benchmark rationale

A biodiversity offset should be designed and implemented in an equitable manner, ensuring that the rights and responsibilities, risks, and rewards associated with the project and its offset are shared fairly among all stakeholders (BBOP 2018). Thus offsets should respect legal and customary arrangements and prioritise the rights of indigenous peoples and local communities recognised at both international and national levels (IUCN 2016; BBOP 2018). Community engagement must follow a free and prior informed consent (FPIC) approach, referring to the right of indigenous peoples to give or withhold their consent for any action that would affect their lands, territories or rights (IFC 2012).

##### Guiding question

16.a) Does the policy ensure that biodiversity offsets are designed and implemented in ways that respect the rights, interests, and well-being of affected communities and promote equitable outcomes?

### Development of the Benchmark for Policy Comparison

This section presents the benchmark developed to evaluate Chilean biodiversity offset policy against international best practices as defined in this paper. The benchmark is composed of 16 principles, each reflecting a core aspect of effective biodiversity offsetting as identified through the literature review (Table 3).

**Table 3:** Application of biodiversity offset principles and policy benchmark in Chile

Principle	Guiding question	Policy source (e.g., article/section)	Excerpt from Chilean policy text	Interpretation
1. Adherence to the mitigation hierarchy	1.a) Does the policy explicitly reference the full mitigation hierarchy (avoid, minimise, restore, offset)?	Guide for Biodiversity Compensation in the EIAS (SEA 2022, p 23)	“The hierarchy of measures is the application sequential application of measures to: 1. Prevent or completely avoid identified impacts on biodiversity. 2. Minimise or reduce identified impacts. 3. Repair the elements of biodiversity affected. 4. Compensate for residual impacts after steps 1, 2 and 3 by replacing the elements at a site other than the one affected.”	The principle is explicitly addressed; the policy clearly requires sequential application of the mitigation hierarchy (hierarchy of measures): 1. avoid, 2. minimise, 3. restore (repair), 4. offset (compensation).
	1.b) Does the policy require demonstrating that avoidance and minimization have been fully considered before offsetting?	Guide for Biodiversity Compensation in the EIAS (SEA 2022, pp 20–21)	“It will only be possible to adequately compensate for residual impacts that remain after measures have been implemented to mitigate and repair the impacts resulting from a project or activity.”	The mitigation hierarchy is clearly embedded in the text, with a structured requirement for following its steps.
2. Biodiversity net gain (BNG)	2.a) Is BNG defined in the policy, and if so, how is it defined?	Guide for Biodiversity Compensation in the EIAS (SEA 2022, p 20)	“Appropriate biodiversity offsetting requires the implementation of measurable actions that compensate for the residual impacts of a project on biodiversity, aiming to produce an alternative and equivalent positive effect in order to achieve zero net loss or, preferably, a net gain in biodiversity.”	The principle is partially addressed; the policy establishes a clear objective of no net loss and expresses a preference for net gain, though the latter is not mandated.
	2.b) Does it specify metrics or methods to demonstrate whether net gain is achieved?	Methodological guide for the compensation of biodiversity in terrestrial and inland aquatic ecosystems (SEA 2023a, p 6)	“The objective of the methodology proposed in this document is to achieve zero net loss of biodiversity.”	The principle is partially addressed; the policy includes a defined methodology for achieving no net loss, while net gain is encouraged but not mandated.
3. Limits to what can be offset	3.a) Are there criteria or thresholds defined to determine when biodiversity loss is unacceptable and cannot be offset?	Guide for Biodiversity Compensation in the EIAS (SEA 2022, p 25)	“The limits for biodiversity offsets are determined by the conditions of irreplaceability and vulnerability.”	Principle is partially addressed; policy acknowledges constraints in some cases but does not clearly define thresholds or non-offsettable impacts.
	3.b) Does the policy provide criteria or guidance to assess when offsets are not feasible or appropriate, including in cases of major uncertainty or high risk?	Guide for Biodiversity Compensation in the EIAS (SEA 2022, p 27)	“The theoretical and practical feasibility of carrying out an appropriate compensation measure should be assessed.”	Principle is partially addressed; policy recognises feasibility constraints, but does not establish clear thresholds for when offsets should not proceed.
4. Additionality	4.a) Does the policy require biodiversity offsets to deliver conservation outcomes beyond those already mandated?	Guide for Biodiversity Compensation in the EIAS (SEA 2022, p 24)	“The results derived from the appropriate compensation actions must be additional to what would have occurred at the site if the measure had not been taken. That is, these actions must result in an improvement in the condition of biodiversity obtained in the offset scenario compared to the no offset scenario.”	There is a clear alignment with the principle; the policy includes a specific obligation to deliver additional conservation benefits.
5. Equivalence/Like-for-like	5.a) Does the policy require offsets to compensate for the full suite of natural and environmental values affected by the project?	Guide for Biodiversity Compensation in the EIAS (SEA 2022, p 24)	“Seeks to ensure that biodiversity elements affected by a project or activity are compensated on the ground by elements of similar characteristics, type, nature, quality and function.”	The principle is fully addressed; the policy explicitly requires offsets the full suite of environmental values affected.
6. Proportionate in size and scale	6.a) Does the policy require that the size and scale of biodiversity offsets be proportionate to the residual impacts?	Guide for Biodiversity Compensation in the EIAS (SEA 2022, p 66)	“The area of compensation accounts for the extent and quality of biodiversity lost”	Principle is clearly addressed; the policy explicitly requires that offset measures be proportional in extent and ecological value to the biodiversity lost.
	6.b) Does the policy provide guidance or criteria for determining that proportionality?	Methodological guide for the compensation of biodiversity in terrestrial and inland aquatic ecosystems (SEA 2023a)	The methodological guide delivers the methodology to define the area to compensate depending on the condition of the biodiversity.	Principle is clearly addressed; the policy provides methodological guidance for determining proportionality.
7. Offsets from earliest stages	7.a) Does the policy require that biodiversity offsets will be considered and planned from the earliest stages of project design and decision-making?	Guide for Biodiversity Compensation in the EIAS (SEA 2022, p 44)	“…the implementation process for the compensation measure should occur as soon as possible during the execution of the project, a factor that should be considered when designing a compensation measure.”	The principle is partially addressed; the policy encourages early consideration of offsets but does not mandate their integration from the initial stages of project planning.
8. Long-term outcomes	8.a) Does the policy require monitoring of offset outcomes over the long term?	Guide for Biodiversity Compensation in the EIAS (SEA 2022, p 19)	“[the measure] should be maintained in the long term, considering the duration of the residual impacts”	The principle is partially addressed; the policy recognises the need to maintain measures in the long term but lacks specificity on enforcement mechanisms or monitoring responsibilities.
	8.b) Does the policy ensure biodiversity gains are maintained beyond the project duration?	Guide for Biodiversity Compensation in the EIAS (SEA 2022, p 44)	“The [compensation] site is adequate, in terms of its administration and management, to ensure that biodiversity elements persist, and their attributes (viability over time) are maintained or enhanced beyond the life of the investment project”	The principle is partially addressed; the policy includes provisions for long-term outcomes, but lacks detail on duration, responsibilities, or mechanisms to ensure ecological persistence over time.
9. Precautionary approach	9.a) Does the policy require precautionary measures when there is uncertainty about potential biodiversity impacts or offset effectiveness?	Guide for Biodiversity Compensation in the EIAS (SEA 2022, p 41)	“Given the uncertainty of predictions, it is important to be conservative in calculations in order to ensure zero net loss.”	The principle is minimally addressed; while the policy acknowledges uncertainty and the need for conservative assumptions, it lacks concrete guidance or enforceable measures to operationalise a precautionary approach.
10. Ecosystem approach	10.a) Does the policy promote an ecosystem approach by considering ecological processes, functions, and interconnections beyond individual species or habitats?	Guide for Biodiversity Compensation in the EIAS (SEA 2022, p 41)	“To consider different levels of biodiversity for the compensation, the characterisation of key biodiversity components at the species, community/habitat and ecosystem/landscape levels must be considered… This characterisation should be carried out for both the site to be negatively impacted and the compensation site(s).”	The policy partially addresses the ecosystem approach by requiring characterisation of biodiversity at multiple ecological levels. However, it does not explicitly address ecological processes or functional interconnections, which are key elements of a full ecosystem approach.
11. Adaptive management and monitoring	11.a) Does the policy require the use of adaptive management strategies based on monitoring results?	Guide for Biodiversity Compensation in the EIAS (SEA 2022, p 46)	“The developer must consider and commit to adaptive management of the sites where compensation is considered, in case monitoring shows that the expected results are not being obtained”	The principle is fully aligned; the policy mandates adaptive management by requiring ongoing monitoring and using the results to inform and adjust compensation measures as needed.
12. Address cumulative, direct and indirect impacts	12.a) Does the policy require consideration of direct, indirect, and cumulative biodiversity impacts in the design and implementation of offsets?	_	_	The principle is not addressed in the policy, as it lacks requirements to consider direct, indirect, and cumulative biodiversity impacts when designing and implementing offsets.
13. Compliance with monitoring and enforcement	13.a) Does the policy include mechanisms to ensure compliance with offset requirements through monitoring, reporting, and enforcement actions?	Guide for Biodiversity Compensation in the EIAS (SEA 2022, p 46)	“It is necessary to include the monitoring of indicators to verify progress towards the desired outcomes, including verifiable milestones with supporting means to prove that they were achieved, at different timeframes.”	The principle is well-integrated, requiring clear mechanisms for compliance through monitoring, reporting, and enforcement of offset.
14. Participatory and transparent approach	14.a) Does the policy promote a participatory and transparent approach by ensuring public access to information?	Guide for Early Citizen Participation (SEA 2023b, p 21)	“The Escazú Agreement highlights the issue of transparency as a guiding principle that relates to other rights. When citizens exercise their right to participation, they need the guarantee of access to environmental information, and with it the obligation to generate and deliver this information, recognising also that, given the resources available, relevant environmental information must be disclosed and disseminated.”	Principle is clearly addressed; the policy explicitly ensures public access to information, supporting a participatory and transparent approach.
	14.b) Does the policy promote a meaningful stakeholder engagement throughout the offset process?	Guide for Biodiversity Compensation in the EIAS (SEA 2022, p 37)	“It is important to identify people and organisations [interested in biodiversity protection or affected by project impacts or even compensation measures] and invite them to participate at an early stage of project development, especially at the design stage of the project and compensation measures, prior to the project’s entry into the EIA System.”	The principle is fully aligned, the policy mandates active involvement of stakeholders throughout the design and execution of biodiversity offsets.
15. Support evidence-based approaches	15.a) Does the policy require the use of scientific evidence, data, and best available knowledge to inform biodiversity offset design, implementation, and evaluation?	Guide for Biodiversity Compensation in the EIAS (SEA 2022, p 39)	“The respect for traditional knowledge requires that it be appreciated in an equitable and complementary manner to the scientific knowledge… fundamental to the sustainable use of biological diversity.”	Policy statements provide strong support for this principle, indicating clear use of scientific evidence and best available knowledge to inform the design, implementation, and evaluation of biodiversity offsets.
16. Equity and rights-based approach	16.a) Does the policy ensure that biodiversity offsets are designed and implemented in ways that respect the rights, interests, and well-being of affected communities and promote equitable outcomes?	Guide for Biodiversity Compensation in the EIAS (SEA 2022, p 39)	“Importance of respecting, preserving, and safeguarding the knowledge, innovations, and practices of indigenous and local communities.”	Principle is partially addressed; the policy acknowledges the importance of equity and community participation but lacks clear mechanisms to ensure rights-based implementation.

## Discussion

Building the benchmarking presented several challenges, since there is no universal agreement on what constitutes best practice principles for biodiversity offsetting. Defining universally accepted principles depends on diverse stakeholder priorities and perspectives, which include conservation goals, social equity considerations and economic interests (Bull et al. 2013; Maron et al. 2016). While this study focuses on ecological and policy dimensions, economic considerations, such as the costs of offsets, incentives for compliance, and allocation of resources, also influence the implementation and effectiveness of biodiversity offsets (Calvet 2015; Simpson et al. 2022). Future research could integrate these economic trade-offs to strengthen decision-making. Additionally, the literature regarding the definition and application of key principles is not consistent and may benefit from intergovernmental negotiation to promote clearer guidance and harmonization. For instance, the concept of NNL is interpreted differently across contexts, largely depending on the reference scenario against which NNL is measured (Maron et al. 2018; Grimm and Köppel 2019). Similarly, the principle of additionality is often ambiguously defined in the literature, leading to varied implementation practices. In some cases, additionality is interpreted narrowly, focusing on direct ecological gains, while in others, it includes broader socioeconomic or policy outcomes (Gardner et al. 2013; Weissgerber et al. 2019). This lack of consistency complicates the establishment of standardised principles, highlighting the need for greater clarity, consensus, and standardisation in the literature. However, effort has been made to address these issues, providing a comprehensive set of principles aimed at harmonising best practices in biodiversity offsetting within projects. The analytical framework developed for the international best practice principles is intended to be globally applicable, serving as a benchmark for evaluating and enhancing biodiversity offset practices across various jurisdictions.

While there is extensive literature on biodiversity offsets and policy, this study provides a novel perspective by directly linking international best practice principles to the Chilean context. Assessing the Chilean biodiversity offset policy against internationally recognized best practice principles demonstrates that this benchmarking approach can effectively evaluate and compare national policies. In the case example evaluated, considerable alignment was identified suggesting that Chile benefits from a good offsets policy, despite some key gaps. While most best practice biodiversity principles are comprehensively addressed in the Chilean policy, certain principles such as BNG, offsets from earliest stages, feasibility of the measures, the precautionary approach principle, and equity and rights-based approach, remain insufficiently integrated. To fully align with global advances in biodiversity conservation (Bull and Brownlie 2017; Maron et al. 2020; Simmonds et al. 2022), Chile’s policy needs to transition towards BNG and fully incorporate these principles. Finally, one principle was completely absent from the national guides, involving addressing cumulative, direct, and indirect impacts, which have not been included within the design of compensation of biodiversity. Although the assessment of cumulative impacts is included in current environmental regulation (MMA 2012), further guidance has been needed to improve conservation outcomes, particularly in relation to biodiversity compensation. In response, Chile is in the process of approving a new regulation, the *Reglamento de compensación de* biodiversidad [Biodiversity compensation regulations], derived from Law N°21,600 *que crea el Servicio de Biodiversidad y Áreas Protegidas (SBAP) y el Sistema Nacional de Áreas Protegidas (SNAP)* [which creates the Biodiversity and Protected Areas Service (SBAP) and the National System of Protected Areas (SNAP)]. This regulation is expected to provide clearer guidance on designing and implementing biodiversity offsets, addressing gaps that have limited the effectiveness of previous compensation measures. While the regulation represents an important step forward, challenges remain in ensuring consistent application integrating ecological, social, and economic considerations. In this context, the benchmarking approach presented in this paper offers a practical tool to evaluate current practices, identify areas for improvement, and support decision-makers in implementing biodiversity compensation measures more effectively.

The existence of a considerable gap between the worldwide implementation of biodiversity offsets and NNL and the supporting evidence for its ecological effectiveness has been described in the literature (zu Ermgassen et al. 2019; Marshall et al. 2024). While biodiversity offsetting has become a widely implemented strategy aimed at mitigating biodiversity loss, there remains a lack of robust, long-term studies demonstrating that these offsets consistently achieve their intended conservation outcomes (Brownlie et al. 2013; Bigard et al. 2017; Bull et al. 2017). Addressing the gap between offsets policy thus requires reinforcing these principles with stronger evidence of more rigorous application.
